# Effect of Preventive *Chlamydia abortus* Vaccination in Offspring Development in Sheep Challenged Experimentally

**DOI:** 10.3389/fvets.2016.00067

**Published:** 2016-08-25

**Authors:** Teresa García-Seco, Marta Pérez-Sancho, Jesús Salinas, Alejandro Navarro, Alberto Díez-Guerrier, Nerea García, Pilar Pozo, Joaquín Goyache, Lucas Domínguez, Julio Álvarez

**Affiliations:** ^1^Centro de Vigilancia Sanitaria Veterinaria (VISAVET), Universidad Complutense de Madrid, Madrid, Spain; ^2^Campus de Excelencia Internacional (CEI) Moncloa, Universidad Politécnica de Madrid (UPM)-Universidad Complutense de Madrid (UCM), Madrid, Spain; ^3^Departamento de Sanidad Animal, Facultad de Veterinaria, Regional Campus of International Excellence ‘Campus Mare Nostrum’, Universidad de Murcia, Murcia, Spain; ^4^MAEVA SERVET S.L., Alameda del Valle, Madrid, Spain; ^5^Departamento de Sanidad Animal, Facultad de Veterinaria, Universidad Complutense de Madrid, Madrid, Spain; ^6^Department of Veterinary Population Medicine, University of Minnesota, Saint Paul, MN, USA

**Keywords:** *Chlamydia abortus*, enzootic abortion, vaccination, challenge, sheep, offspring development

## Abstract

Ovine enzootic abortion, caused by *Chlamydia abortus*, leads to important economic losses worldwide. In addition to reproductive failures, infection may impact lamb growth during the first weeks after birth, yet this effect has not been well characterized. Vaccination can help to control the disease but variable efficacy values have been described, possibly related with factors associated with the host, the vaccine, the parameter used for efficacy determination, and the challenge conditions. In this context, we evaluated the efficacy of an inactivated standard commercial vaccine and a 1/2 diluted dose in pregnant sheep challenged with *C. abortus* by examining multiple indicators of vaccine effect (including incidence of reproductive failures, bacterial excretion, and evolution of weight gain of viable lambs during the first month of life). Three groups of ewes [control non-vaccinated, C (*n* = 18); vaccinated with standard dose, SV (*n* = 16); and vaccinated with 1/2 dose, DV (*n* = 17)], were challenged approximately 90 days post-mating and tested using direct PCR (tissue samples and vaginal swabs) and ELISA (serum) until 31 days post-reproductive outcome. There were not significant differences in the proportions of reproductive failures or bacterial shedding after birth/abortion regardless the vaccination protocol. However, a beneficial effect of vaccination on offspring growth was detected in both vaccinated groups compared with the controls, with a mean increase in weight measured at 30 days of life of 1.5 and 2.5 kg (*p* = 0.056) and an increase in the geometric mean of the daily gain of 8.4 and 9.7% in lambs born from DV and SV ewes compared with controls, respectively. Our results demonstrate the effect of an inactivated vaccine in the development of the offspring of *C. abortus*-infected ewes at a standard and a diluted dose, an interesting finding given the difficulty in achieving sufficient antigen concentration in the production of enzootic abortion of ewes-commercial vaccines.

## Introduction

*Chlamydia abortus* is an obligate intracellular, Gram-negative bacterium that belongs to the family *Chlamydiaceae* (1). Ruminants are its main hosts, and infection leads to a disease, known as enzootic abortion of small ruminants, characterized by reproductive failures (late-term abortion, neonatal death, and premature offspring). In addition, *C. abortus* infection can be responsible for weak/low birth-weight lambs (2, 3) and can affect early lamb development (4), although the contribution of this key aspect on the full impact caused by the disease has not been characterized in detail. The disease has a worldwide distribution and is recognized as a major cause of economic loss in most sheep-rearing regions [including Northern Europe, North America, and Africa (5)], with the exception of Australia and New Zealand (6), and is considered the main cause of infectious abortion in sheep [enzootic abortion of ewes (EAE)] in the United Kingdom (7) and Switzerland (8). In addition, *C. abortus* may be a zoonotic agent that can induce reproductive disorders in pregnant women (5).

In EAE-infected flocks, ewes shed high amounts of *C. abortus* in vaginal discharges during abortion/parturition and thus placenta, fetuses, and lamb coats born to infected ewes are heavily contaminated. Susceptible animals can become easily infected by the ingestion of contaminated material (9) or the inhalation of aerosols (10). EAE-affected ewes will develop an effective post-infection immune response preventing future *C. abortus*-induced abortions (11), but may still shed the bacteria in the following estrus, contributing to the maintenance of the infection in the flock. The reproductive failure rate in a newly infected naive sheep flock is around 30% (up to 60% in goat herds), whereas in endemically infected flocks, prevalence is around 10% (12). The most effective strategy for prevention of EAE is the immunization of susceptible individuals (9). Vaccination strategies aim to trigger a protective immune response similar to that occurring after abortion in EAE-infected ewes (13). Nowadays, currently available vaccines against *C. abortus* include inactivated and live-attenuated products (14). The live temperature-sensitive attenuated vaccine has been shown to offer good protection against *C. abortus*-induced abortion in field trials (9, 15). However, given the zoonotic nature of the microorganism, and the recent description of outbreaks of abortions in vaccinated flocks in which a vaccine strain was implicated (16, 17), this commercially available attenuated vaccine pose safety concerns. By contrast, inactivated vaccines, prepared from egg-grown strains or cell cultures, have been widely used in the control of EAE since its infection nature was discovered in the 1950s, and its efficacy has been well documented (4, 18–21), leading to its recommendation as a useful control option by the OIE (14). However, although inactivated vaccines may reduce the abortion rate, they do not completely prevent bacterial shedding and reproductive failures may still occur (9). In addition, the efficacy of inactivated vaccines is highly variable (9), with reports of occasional EAE outbreaks in vaccinated flocks (22–24).

Vaccine efficacy trials are usually focused on assessing the impact of vaccination on the abortion rate and bacterial excretion, as well as parameters related to viable lambs at birth [lamb weight and kilograms of lamb per ewe (4)]. However, as discussed earlier, the effect of vaccination in the early development of viable offspring has not been thoroughly studied, despite being a key aspect contributing to the full economic impact of EAE.

In this context, the aim of the present study was to assess the efficacy of a full and a diluted dose of a commercial inactivated vaccine against EAE on eliciting a protective immune response against a subsequent *C. abortus* challenge in pregnant sheep, considering not only classical microbiological and clinical parameters but also for the first time a detailed evaluation of the weight development of the offspring.

## Materials and Methods

### Animals and Experimental Design

A total of 80 primiparous 10-month age crossbreed “Merino” ewes were randomly selected from a flock without history of abortions located in Central Spain. This sample size was selected in order to achieve at least 20 pregnant sheep in each of the vaccinated groups of study (as required by the European Pharmacopoeia for ovine vaccines against other infectious abortifacient diseases) (25). All animals tested negative for *C. abortus, Salmonella enterica* serovar Abortusovis, *Brucella* spp., *Toxoplasma gondii, Coxiella burnetii*, and Maedi-Visna virus before being included in the study. Sheep were then randomly allocated in three experimental groups: SV (standard vaccine, *n* = 25), DV (diluted vaccine, *n* = 29), and C (control group, *n* = 26). Finally, the number of pregnant sheep that we obtain was 16 in SV group, 17 in DV, and 18 in group C. Food and water were provided *ad libitum* throughout the study. All husbandry practices and animal procedures were authorized by the scientific and animal experiments committee of Complutense University of Madrid (98/2012) and the Animal Research Committee from the Madrid Region (10/176335.9/11).

### Vaccination Protocol

Animals from Group SV were vaccinated with a commercial bivalent vaccine against *C. abortus* (concentration of 10^7.8^ egg lethal dose 50) and *Salmonella enterica* serovar Abortusovis (concentration of 2 × 10^9^ colony-forming units) in oiled adjuvant. Sheep belonging to group DV were immunized with a 1/2 dilution of the commercial vaccine. Finally, ewes from group C were inoculated with 0.85% saline sterile solution (control group). In the three groups, the vaccination protocol consisted in a 2-ml first dose applied at day 0 and a booster of 2-ml applied 29 days later. Vaccines and saline solution were inoculated subcutaneously in the axillary region.

### Breeding Protocol

Sixteen days after inoculation of the first dose [16 days post-vaccination (d.p.v.)], ewes were mated with *C. abortus* seronegative rams. Previously, estrus of the sheep had been synchronized using vaginal sponges with cronolone (Chronogest^®^, MSD, Salamanca, Spain) and a intramuscular injection of mare serum gonadotroping (Folligon^®^, MSD, Salamanca, Spain), according to the manufacturer’s instructions. Pregnancy was confirmed by transabdominal ultrasound at 60 d.p.v. All pregnant sheep (16 ewes in group SV, 17 ewes in group DV, and 18 ewes in group C) were placed in different barns depending on the experimental group.

### Experimental Challenge

After 103 d.p.v. (87 days after breeding), all animals were challenged using 2 ml of a 5 × 10^6^ inclusion-forming units/ml of *C. abortus* (dose per animal: 1 × 10^7^ inclusion-forming units) inoculated subcutaneously. The challenge inoculum was prepared from a culture of the pathogenic *C. abortus* AB7 strain grown in yolk sacs of developing chicken embryos. Titers of inocula were established by culture in MCoy cell line and subsequent count of inclusion-forming units, as previously described by Buendía et al. (26). The original inoculum was stored at −80°C until the challenge, when it was reconstituted in sterile phosphate-buffered saline (PBS) solution.

### Sampling Strategy

Serum samples were collected at 0 and 29 d.p.v., biweekly until the beginning of the abortions/parturitions and weekly since then to the end of the study. Rectal temperature between days 0 and 6 post-challenge was measured daily. Vaginal swabs from all ewes were collected within the first 3 days post-parturition/abortion and then weekly for at least 21 days after the reproductive outcome using commercial sterile swabs with transport media for *Chlamydia* (Deltalab, Barcelona). Samples from spleen, liver, lung, and stomach content (as well as cotyledons, when available) from all aborted/non-viable lambs were collected immediately after delivery. Vaginal swabs and tissue samples were processed immediately after collection or stored at −80°C.

### Laboratory Tests

#### PCR Detection of *C. abortus*

Vaginal swabs were resuspended on 1.5 ml of sterile PBS and subjected to DNA extraction using QIAamp MinElute Virus Spin Kit (Qiagen, Las Rozas, Madrid) according to the manufacturer’s instructions. Tissue samples from fetuses and dead lambs were processed using the DNEasy Blood and Tissue Kit (Qiagen, Las Rozas, Madrid), according to the manufacturer’s instructions. DNA extracted from all samples was analyzed using a real-time PCR, as previously described (27).

#### Serology

Serum was recovered from blood samples and analyzed using an ELISA commercial kit [ID Screen *Chlamydophila abortus* indirect multispecies (IDvet, Grabels, France)] according to the manufacturer’s instructions. This test expresses the result of a sample as the percentage of the optical density (%OD) value obtained in the positive controls adjusted by background readings as follows:

$$\%\text{OD}=\frac{{\text{OD}}_{\text{sample}}-{\text{OD}}_{\text{negative control}}}{{\text{OD}}_{\text{positive control}}-{\text{OD}}_{\text{negative control}}}\times 100$$

Samples with %OD values >60% were classified as positive.

### Statistical Analysis

Vaccine effect was evaluated by comparing the values of the following indicators in the different study groups:

1. Post-vaccination and post-challenge serological responses: ELISA results in the different sampling points after the vaccination/challenge were compared using the chi-square and *Z*-test for comparison of proportions of reactors and the Mann–Whitney and Kruskal–Wallis test with *p*-values adjusted using the Bonferroni method. Qualitative and quantitative ELISA responses were also compared depending on the reproductive outcome (reproductive failure with positive *Chlamydia* PCR result/negative PCR deliveries).
2. Daily rectal temperature between days 0 and 6 post-challenge (d.p.c.): changes in the rectal temperatures before (0 d.p.c.) and after challenge (1–6 d.p.c.) were compared using Wilcoxon test.
3. Number of reproductive failures (abortion/stillbirth lambs/weak lambs that died in the first 5 days of life) associated with *C. abortus* infection (those in which DNA from the pathogen was detected by real-time PCR in offspring tissues, placenta, and/or vaginal swab from the mother). The relative risk of reproductive failure associated with *C. abortus* infection in each vaccinated group compared with the controls was calculated using the MedCalc Statistical Sofware bvba 15.8 (Microsoft Partner, Ostend, Belgium) (28).
4. Percentage of shedder animals, defined as those from samples in which *C. abortus* was positive (organs from fetuses/non-viable offspring and/or vaginal swabs): proportions of positive ewes were compared using the *Z*-test for comparison of multiple proportions adjusted by the Bonferroni method.
5. Total weight of lambs born per ewe: the mean total weight of lambs born from ewes in each group recorded at birth and at 30 days of life as well as the total gain achieved during those 30 days were compared using a mixed model including mother as a random effect and twins as a fixed effect.
6. Daily weight gain of lambs from ewes of each group during the first 31 days of life (offspring development): to evaluate the effect of vaccination on the daily weight gain in the lambs from animals receiving the standard (SV group) and diluted (DV group) doses compared with the controls, a multivariable Bayesian regression model was fit, such that:

$$y_{\text{i,j}}\sim N(\mu_{\text{i,j}},\sigma )$$

where *y*_i,j_ represents the log-transformed weight from lamb i recorded in day j during the first 31 days after birth, and

$$\mu_{\text{i,j}}=\alpha_{\text{i}}+{\text{β}}_{0}+{\text{β}}_{1}X_{\text{i1}}+{\text{β}}_{2}X_{\text{i}2}+\ldots \beta_{\text{k}}X_{\text{ik}}$$

with *X*_ik_ denoting the kth covariate in lamb i with the corresponding regression parameter β_k_ and α_i_ representing the random effect corresponding to lamb i (included to account for the lack of independence between observations recorded from the same animal). The effect of the number of days after lambing (1–30), group of study of the mother (SV, DV, and C), type of pregnancy (single or twin) and the weight registered the day of birth were evaluated as fixed effects in the model. Non-informative Gaussian functions with mean 0 and variance 1,000 were used as prior distributions for the regression coefficients β_0_ and β_k_, and α_i_ was assumed to follow a normal distribution *N*(μ_α_, σ_α_), where μ_α_ followed a normal distribution as described before and σ_α_ followed a uniform distribution (0.01, 100).

All tests for indicators 1–5 were performed using the software SPSS 20 (IBM, New York, NY, USA) (29) except when stated otherwise, and interpreted considering a *p*-value of 0.05 to determine statistical significance. The normality of the quantitative values was assessed using the Kolmogorov–Smirnov test before further analyses were carried out.

Models for indicator 6 were run in WinBUGS 1.4 using the package R2WinBUGS from the software R 3. 0. 3 (30). Each model was evaluated using three Monte Carlo Markov Chains (MCMC) and convergence and mixing was assessed visually and more formally using the Gelman–Rubin *Rˆ* statistic (31, 32). Models were run for 5,000 iterations after discarding the first 2,500 burn-in samples for obtaining the posterior estimates, and autocorrelation was eliminated by thinning the samples by collecting one in 10 consecutive samples. The best model was selected by exploring the combination of variables and two-way interactions that best explained the observed data based in Deviance Information Criterion (DIC) (33). Model checking was performed by comparing the observed data with the posterior predictive distribution of the observations generated using the fitted model, as previously described (34).

## Results

### Post-Vaccination and Post-Challenge Serological Responses

**Figure 1:**
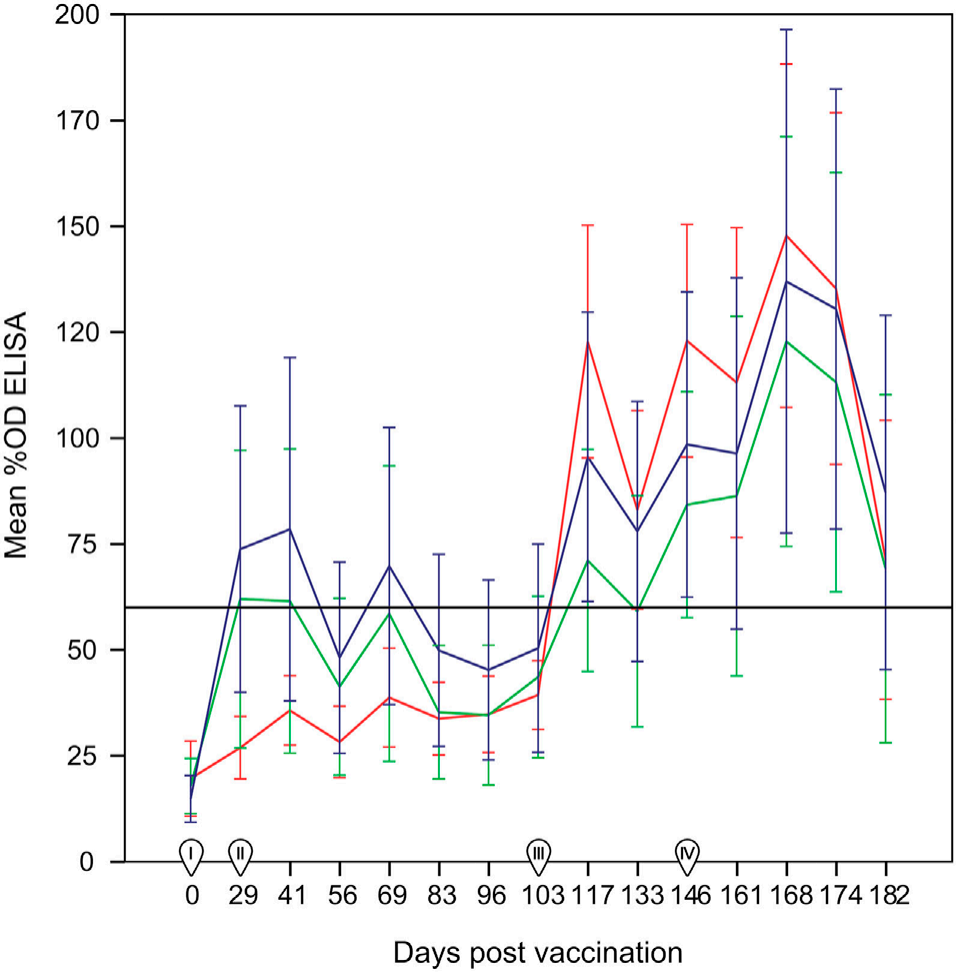
**Percentage of optical density (%OD) in the ELISA test per group (mean ± 95% confidence interval) between 0 and 182 days post-vaccination**. Red line: group C (non-vaccinated control group). Blue line: Group SV (standard dose vaccine). Green line: group DV (1/2 dose vaccine). The horizontal continuous line indicates the cut-off of the ELISA test (60%OD). I: day of administration the first vaccine dose subcutaneously. II: day of administration the second vaccine dose subcutaneously. III: day of experimental infection with a dose of 1 × 10^7^ inclusion-forming units of *C. abortus* strain AB7, applied subcutaneously. IV: beginning of reproductive events. Error bars: 95% confidence interval.

**Figure 2:**
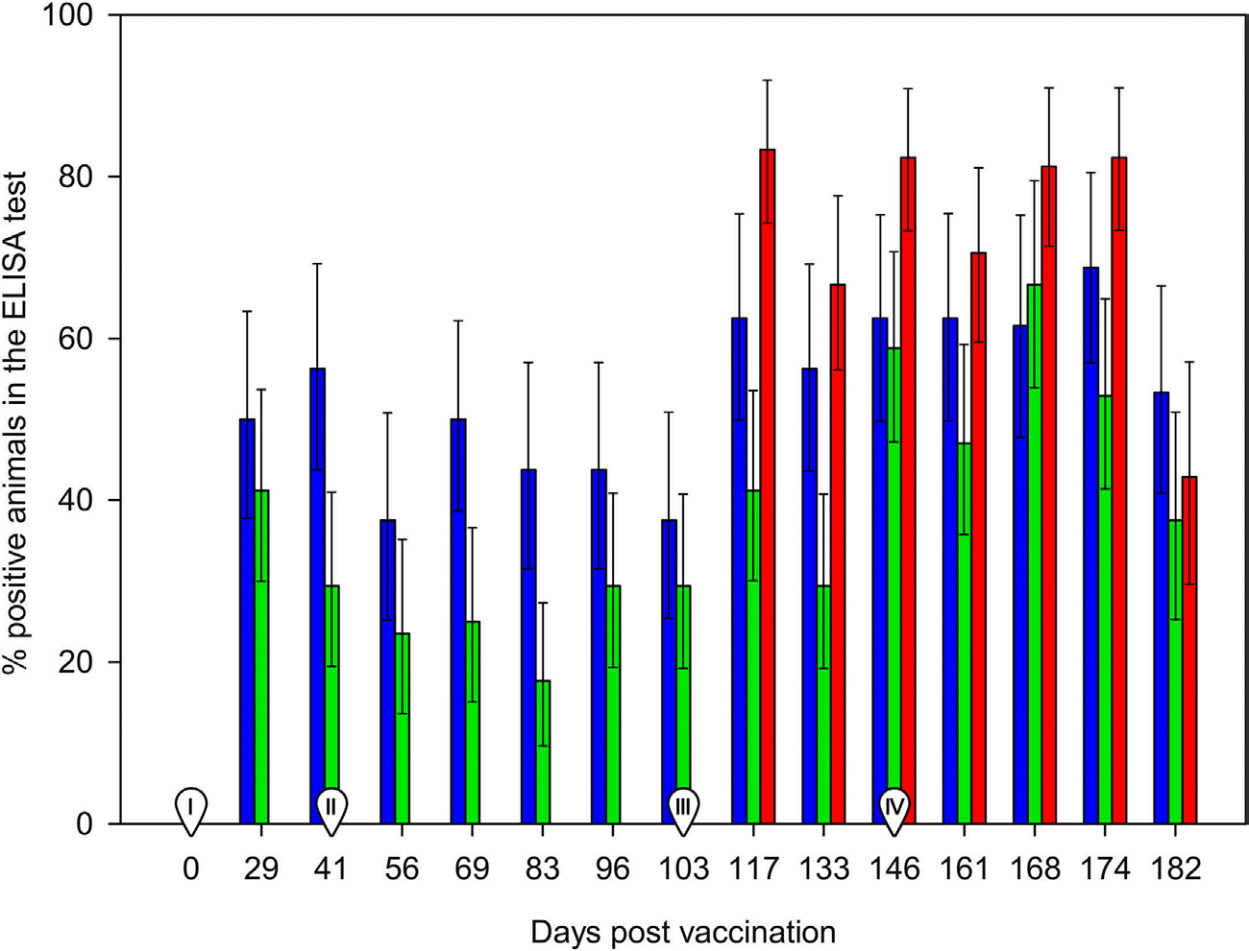
**Percentage of ELISA positive animals between 0 and 182 days post-vaccination**. Red bars: group C (non-vaccinated control group). Blue bars: Group SV (standard dose vaccine). Green bars: group DV (1/2 dose vaccine). Error bars indicate SEs. I: day of administration the first vaccine dose subcutaneously. II: day of administration the second vaccine dose subcutaneously. III: day of experimental infection with a dose of 1 × 10^7^ inclusion-forming units of *C. abortus* strain AB7, applied subcutaneously. IV: beginning of reproductive events.

The day of vaccination all animals were seronegative in the ELISA test. Ewes from the control group (non-vaccinated) remained negative until the challenge, while positive responses were already recorded at 29 d.p.v. (first sampling post-vaccination) in both SV and DV groups (Figure 1). Most of the animals seroconverted on day 29 (8/16 in the SV and 7/17 in the DV group), which was also the day when the largest proportion of reactors and higher %OD values before the challenge for group DV were recorded (41%, 7/17). By contrast, in the SV group, the overall maximum number of seropositive animals and %OD values were observed 2 weeks later (41 d.p.v., 56%, 9/16) (Figure 2; Table S1 in Supplementary Material). The proportion of reactors decreased 37% (6/16) and 29% (5/17) in SV and DV groups, respectively, prior to the challenge (performed at day 103 d.p.v.) (Figure 2; Table S1 in Supplementary Material). Seven and four animals in the DV and SV groups did not seroconvert prior to the challenge (Table S1 in Supplementary Material), respectively. Proportion of positive reactors and %OD between vaccination and challenge were higher in the SV group than in the DV and the controls (Figures 1 and 2; Table S1 in Supplementary Material), although no significant differences in any sampling day were observed between vaccinated groups (*p* > 0.05).

After the challenge and before the beginning of reproductive outcomes, the highest proportion of reactors in the three groups was observed at 14 d.p.c. (117 d.p.v.), ranging from 7/17 in DV group (significantly lower than in the C group, *p* < 0.05) to 10/16 and 15/18 in the SV and C groups (Figure 2, Table S1 in Supplementary Material). No significant differences in the proportion of reactors were detected in subsequent samplings. Animals from group C showed the highest %OD values (Figure 1), although differences were only significant 14 d.p.c. (117 d.p.v) (Kruskal–Wallis test, *p* = 0.03), when differences between the C and DV groups were detected (Pairwise-comparison with Bonferroni adjustment, *p* = 0.024). The four ewes from the SV and four out of the seven ewes in the DV that did not seroconvert after vaccination were also never positive after the challenge. From the group of ewes that were positive at some stage after the vaccination, 16 were positive in the first sampling after the challenge, and only 4 became positive at some point afterward (Table S1 in Supplementary Material).

**Figure 3:**
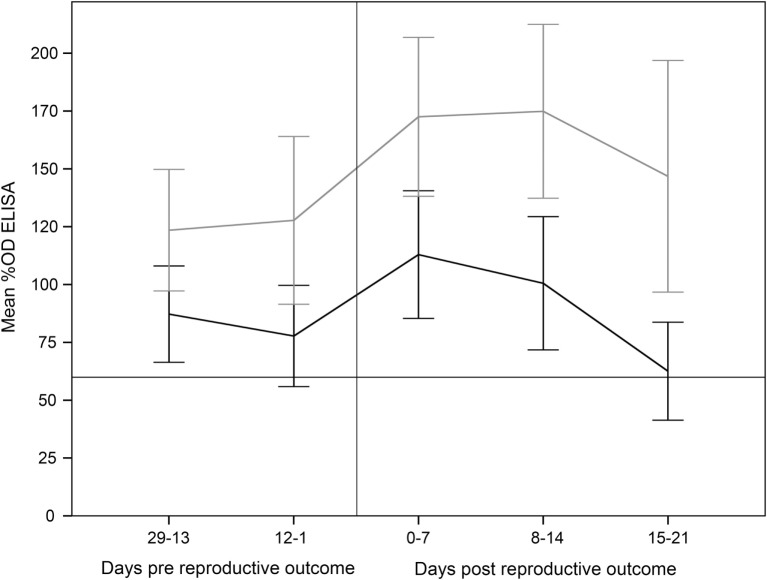
**Percentage of optical density (%OD) in the ELISA test (mean ± 95% confidence interval) from 29 days prereproductive outcome to 21 days after reproductive outcome, depending on the association of reproductive failure with *C. abortus* infection**. Error bars: 95% confidence interval. Gray line: ewes with reproductive failure associated with *C. abortus*. Black line: ewes with reproductive outcome not associated with *C. abortus* infection.

**Figure 4:**
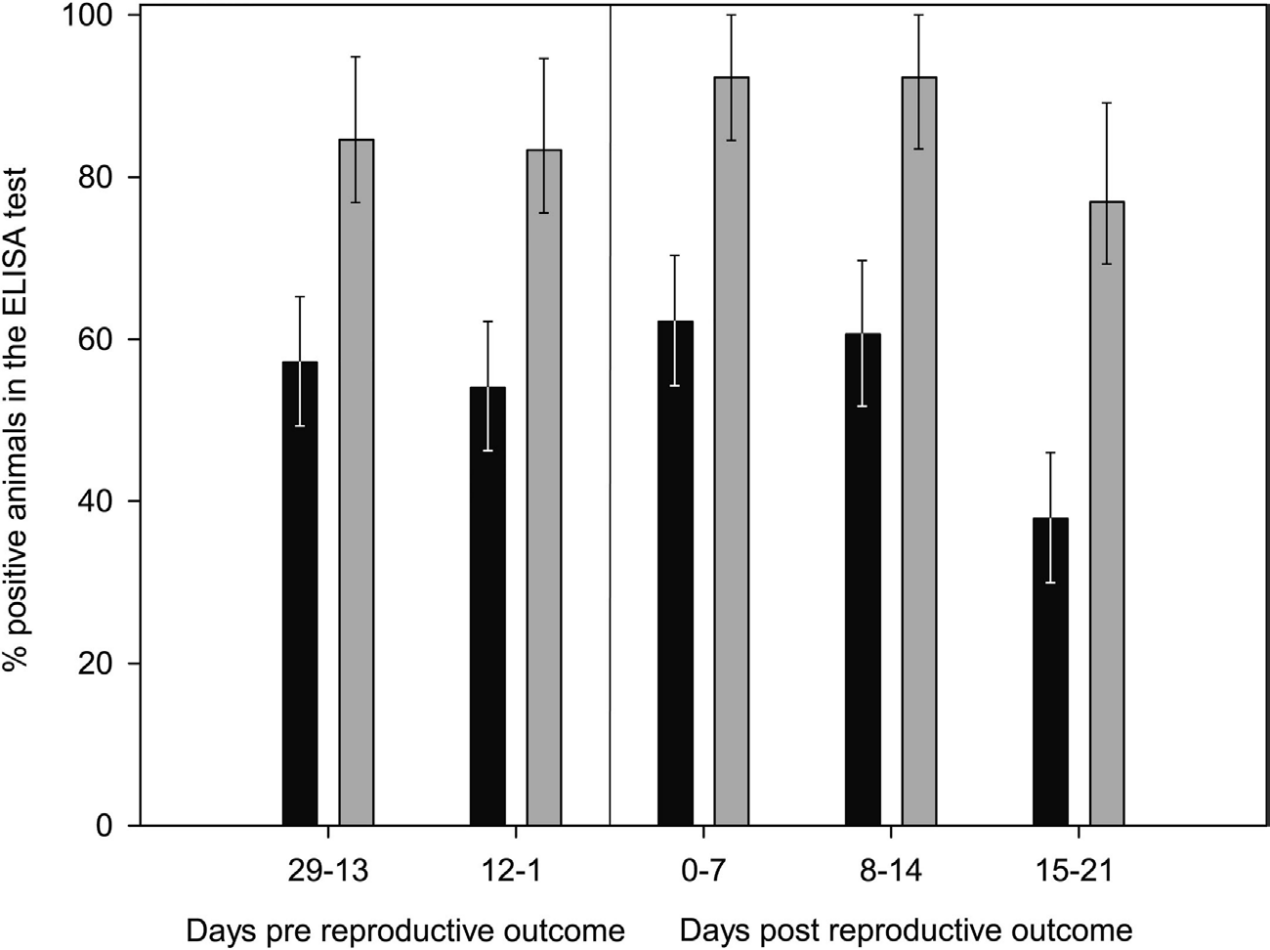
**Evolution of percentage of positive animals in the ELISA test from 29 days prereproductive outcome to 21 days after reproductive outcome, depending on the association of reproductive failure with *C. abortus* infection**. Gray bars: ewes with reproductive failure associated with *C. abortus*. Black bars: ewes with reproductive outcome not associated with *C. abortus* infection. Error bars indicate SEs.

Finally, the increase in the %OD after the reproductive outcomes was observed regardless the study group, with no significant differences in the qualitative or quantitative ELISA results between groups detected in any sampling day (Figures 1 and 2; Table S1 in Supplementary Material). However, if ELISA results were sorted in relation to the reproductive outcome (days post-reproductive outcome, d.p.r.) significantly higher %OD values were observed for samples collected within 1 week before and within 1, 2, and 3 weeks after the reproductive outcome in animals with a positive PCR result (Figure 3). Percentage of reactors was also significantly higher (*p* < 0.05) for PCR-positive animals for samples collected within 15–21 (10/13 vs. 14/37) d.p.r. (Figure 4). No differences in the ELISA results depending on the study group for samples collected in any period before or after the reproductive outcome were observed.

### Daily Rectal Temperature

Comparison of temperatures measured on the day of the challenge and 1 d.p.c. revealed a mild though significant increase after the challenge for the DV (median increase of 0.3°C, *p* = 0.02) and C (median increase of 0.52°C, *p* = 0.006) groups while this was not observed for the SV group (median increase of 0.25°C, *p* = 0.1). No further significant increases of temperature were detected 2–6 d.p.c. compared with the temperature on the day of the challenge (data not shown).

### Reproductive Failures Associated with *C. abortus*

One ewe from group C with a twin pregnancy died during delivery (lamb-tissues and vaginal swabs were PCR-negative) due to a dystocia and was excluded from analysis of proportions of reproductive failures. Results of the reproductive events and their PCR results per study group from the remaining animals are summarized in Table 1. A total of 4/17, 4/16, and 3/17 ewes from the SV, DV, and C groups, respectively, had a *C. abortus*-associated reproductive failure (Table 1). No significant differences in the relative risk of reproductive failure associated with *C. abortus* between the three groups or vaccinated vs. non-vaccinated groups were observed (*p* > 0.05). While *C. abortus* DNA was detected in all abortions regardless the experimental group, it was only found in 3/5, 0/1, and 1/2 weak lambs born from mothers in groups SV, DV, and C, respectively. Three lambs born from ewes belonging to the SV, DV, and C groups (one lamb per ewe) died 17, 30, and 6 days post-birth (with positive PCR results for lambs born from SV and DV ewes and negative results for the lamb born from the C ewe) but were not classified as weak for consistency with the pre-established definition.

**Table 1:** **Summary of reproductive events and *C. abortus* real-time PCR results in tissues from fetuses/dead lambs and vaginal swabs from ewes after challenge observed in each experimental group**.

		No. ewes	No. ewes with + PCR of dead offspring and/or VS at some point	Positive vaginal swab PCR results					
				0–3 DPR	4–14 DPR	15–22 DPR	23–30 DPR	0–30 DPR (total group)	
Group SV	Abortion/stillbirth	1	1	1/1	0/1	1/1	0/1	1/1	(7/16)
	Weak lambs	5	3	2/5	1/4	2/5	0/5	2/5	
	Healthy lambs	10	5	2/9	2/10	1/10	1/8	4/10	
Group DV	Abortion/stillbirth	3	3	2/3	2/3	1/3	1/2	2/3	(5/17)
	Weak lambs	1	1	0/1	1/1	0/1	0/1	1/1	
	Healthy lambs	13	3	1/13	2/13	2/12	0/11	2/13	
Group C	Abortion/stillbirth	2	2	1/2	1/2	1/2	1/2	1/2	(4/17)
	Weak lambs	1	1	0/1	0/1	0/1	0/1	0/1	
	Healthy lambs	14	3	3/13	2/14	2/14	0/11	3/14	
Total (%)		50	22	12/48 (23%)	11/49 (22%)	10/49 (20%)	3/42 (7%)	16/50 (32%)	

*DPR, days post-reproductive outcome; Group SV, standard dose vaccine; Group DV, 1/2 dose vaccine; Group C, control ewes, non-vaccinated VS, vaginal swab. In the fraction, numerator indicates number of PCR-positive vaginal swabs and denominator indicates number of tested vaginal swabs*.

Overall, a total of 13, 15, and 20 viable lambs were born from 12 SV, 14 DV, and 15 C ewes, respectively. Twin gestations were observed in all experimental groups [*n* = 3 in group SV (one of these a parturition of three lambs), *n* = 2 in group DV and *n* = 6 in group C].

### Proportion of Shedders per Vaccination Group

No significant differences in the proportion of ewes shedding *C. abortus* (as determined by vaginal swabs and samples from dead offspring) depending on the study group or vaccination status (vaccinated vs. non-vaccinated) were detected (*p* > 0.05). Approximately 25% of the animals tested positive within the first 3 d.p.r and in the following 22 days, and this proportion decreased in subsequent samplings up 7% approximately 4 weeks after the reproductive outcome (Table 1).

### Total Weight of Lambs Born per Ewe

The mean total weight of lambs (15/17 in group SV, 14/16 in group DV, and 18/21 in group C) at 30 days of life was higher for the vaccinated groups compared with the control group (Table 2), but while lambs from the DV were slightly larger at birth those born from SV ewes were larger at 30 days of life (Table 2). The effect of the vaccination group of the ewe was, however, only borderline significant (*p* = 0.056) for the weight recorded at 30 days of life.

**Table 2:** **Medium weight recorded at birth and 30 days of life and overall mean weight gain (in kilograms ± SD) measured in lambs born from ewes in the SV (standard dose vaccine), DV (1/2 dose vaccine), and C (control) groups**.

	Medium weight of lambs born per ewe at birth	Medium weight of lambs at 30 days post-partum	Medium weight gained in the 30 first days of life
Group SV	3.21 ± 1.10	9.28 ± 2.00	5.43 ± 1.53
Group DV	3.31 ± 0.79	8.21 ± 2.85	4.82 ± 2.29
Group C	2.90 ± 0.73	6.69 ± 2.30	3.87 ± 2.10

### Daily Weight Gain within the First 31 Days of Life

**Figure 5:**
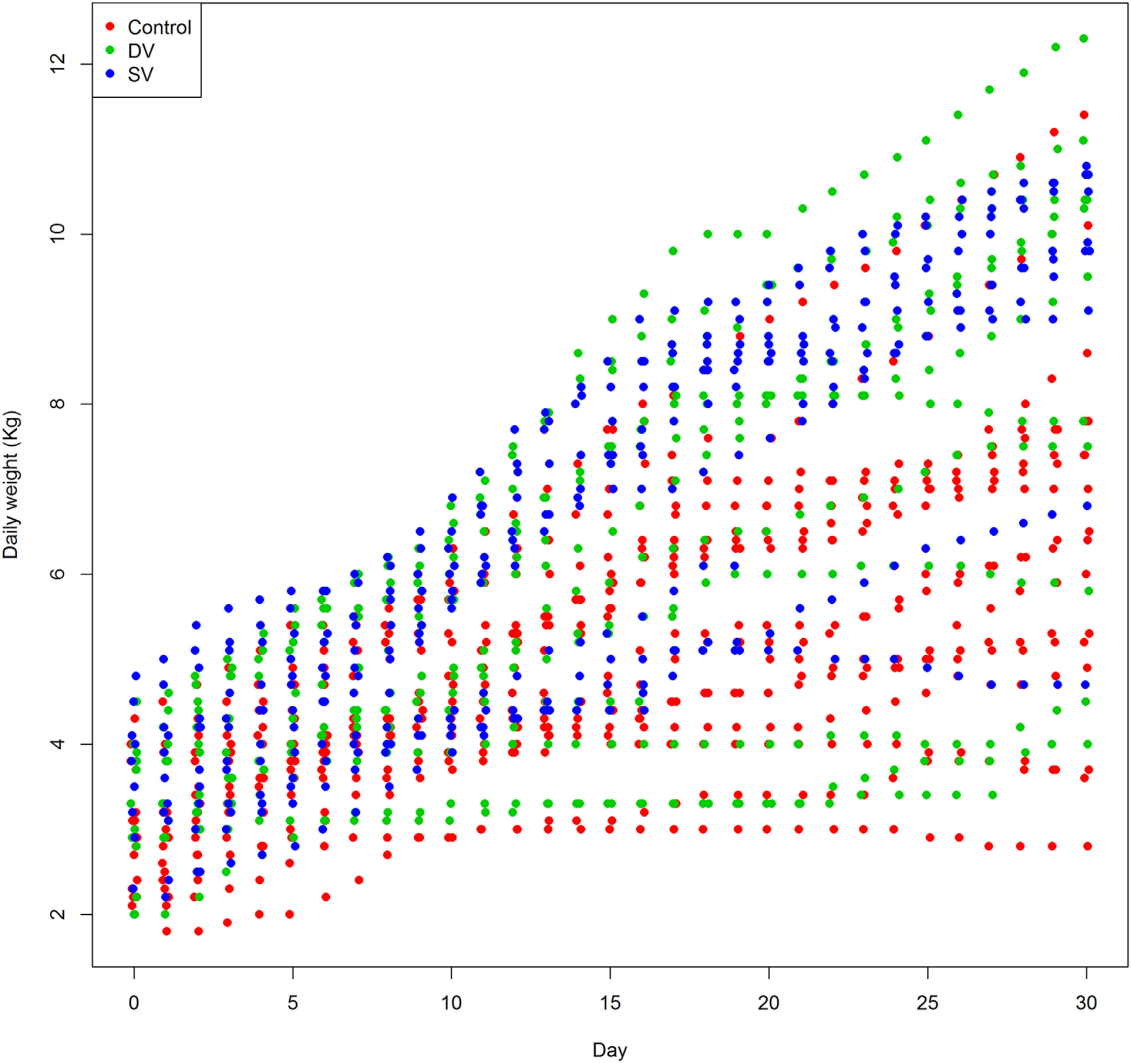
**Daily weight recorded in 48 lambs born from ewes in the control (red dots), DV (1/2 dose vaccine, green dots), and SV (standard dose vaccine, blue dots) in the first 30 days of life**.

The weight gain in lambs born from ewes in groups SV and DV was greater than in lambs from group C, thus suggesting a relative but significant effect of vaccination on the weight of the offspring in the first 30 days of life (Table 3; Figure 5). An increase of the geometric mean of the non-transformed weight of 9.7% (95% posterior probability interval 6.6–12.8) and 8.4% (95% posterior probability interval 5.4–11.5) was observed in lambs born from ewes receiving the standard (SV group) and diluted vaccination dose (DV group), while no differences between the weights recorded in lambs born from the two vaccinated groups were found. A large variation in the values estimated for the lamb-random effect that included the effect of the day of measurement was observed, highlighting the individual-level variability throughout the 30 days of measurements (Figure S1 in Supplementary Material).

**Table 3:** **Median parameters of the model measuring the association between the log-transformed weight recorded in 47 lambs during their first 30 days of life and the lamb-related variables**.

Variable	Value	Estimate	95% posterior probability interval		Rhat
Intercept		1.010	0.978	1.043	1.0006
Vaccination group	DV	0.080	0.052	0.109	1.0034
	SV	0.093	0.064	0.121	1.0054
Type of pregnancy	Twin	−0.144	−0.120	−0.169	1.0004
Log of weight at birth		0.233	0.210	0.258	1.0022

*SV, standard dose vaccine; DV, 1/2 dose vaccine*.

Final models converged adequately as demonstrated by visual assessment of the MCMC, effective sample sizes, and the *Rˆ* value <1.1 found for all parameters, and predictive checking revealed an adequate fit of the model to the data.

## Discussion

*C. abortus*, etiological agent of EAE, is one of the most important pathogens for small ruminant production worldwide in terms of its economic impact. In the United Kingdom (the major sheep producer in Europe), alone the economic burden imposed by EAE was estimated to be around £20 million/year (US$28 million) (35). Control of the disease is usually pursued using live or inactivated vaccines, but vaccine efficacy is variable and knowledge on the efficacy of currently available vaccine is limited. In the present study, the protection of a standard (group SV) and a 1/2 diluted dose (group DV) of a commercial vaccine against a *C. abortus* challenge was evaluated in pregnant sheep. Group DV was included in the study in order to evaluate vaccine efficacy at reduced doses, as previously described (36), since one of the major economic constraints in the production of EAE-commercial vaccines is the antigen concentration necessary to ensure an adequate protection (37). Although no differences in the incidence of abortions between the vaccinated and the control groups were observed, a protective effect of the vaccine on the lamb weight gain during the first month of life of the viable lambs was detected using a model that allowed the analysis of longitudinal data. This effect was observed regardless of the vaccine dose (standard or diluted) used.

Lack of differences between vaccinated and control groups had been reported also in previous studies using other commercial inactivated vaccines (4, 12, 38, 39). However, the boost in the serological response detected by the ELISA in both vaccinated groups (Figure 1) highlights the immunogenicity of *C. abortus*-inactivated vaccines, evident between 29 and 41 d.p.v., as previously reported (40–42) [showing group SV a higher response than group DV as observed in Wilsmore et al. (3)], although different inactivated vaccines have been also reported to induce a scarce (43) or null (4) serological response.

Experimental challenge induced a mild but significant increase in temperature 1 d.p.c. in the C and SV groups and a general seroconversion in all experimental groups, as previously described (Figure 1) (3, 4, 42, 44). A similar phenomenon (seroconversion) was observed in most animals after parturition/abortion in agreement with previous reports (Figure 1) (4, 36, 44, 45). Significantly higher %OD values in animals with a reproductive failure associated with *C. abortus* compared with the rest of the ewes (Figure 3). This increase in the antibody levels when *C. abortus*-associated reproductive failure occurs has been reported before (3, 36, 46, 47) and could be associated with the development of protective immunity against subsequent abortions (5).

Proportion of *C. abortus*-associated reproductive failures and ewes shedding *C. abortus* post-reproductive outcome (in vaginal swabs and/or their fetuses/dead lambs) were limited in all groups (<45 and <56%, respectively, Table 1) compared with previous studies that reported rates between 40 and 90% for reproductive failure (4, 48–52) and 40 and 100% for *C. abortus* shedding (4, 20, 42, 50). This finding could be explained at least in part by several factors. First, the moment of the experimental infection during the pregnancy has been demonstrated to impact the ability of the ewe to control it (11, 53, 54); here, the challenge was performed at approximately 85 ± 8 days of pregnancy in contrast with previous reports in which it took place between 60 and 75 days of pregnancy, considered the most susceptible period (2, 7, 42, 44, 46, 55). A challenge performed later during pregnancy could lead to a lower degree of colonization of the placenta and a milder infection and, ultimately, a lower level of excretion (thus being more difficult to detect by PCR) and a lower proportion of abortions (9, 12) as supported by results based on a late (90–105 days post-mating) challenge (4, 23, 39). Second, samples from the placentas (the target tissue for *C. abortus* multiplication) (56) were only available from three ewes, what could have led to a more limited sensitivity of the direct PCR, a technique that has also been reported to be less sensitive than culture (57). Finally, lack of vaginal swabs collected at delivery/abortion for some animals that were sampled in days 1–3 post-reproductive outcomes may have contributed to the underdetection of animals with a limited and brief bacterial excretion after delivery.

Antibody responses are thought to play a role preventing *C. abortus* reinfection, although their protective effect against EAE is not fully understood (54). In fact, in the present study, 10 ewes (8 vaccinated and 2 control) with different reproductive outcome (2 reproductive failures, 7 normal parturitions and the sheep from group C died during delivery, of which 3 were positive in the PCR) never seroconverted during the study, demonstrating that serological responses do not always correlate with *C. abortus* previous contact, reproductive failure, or vaccine efficacy, in agreement with previous findings (11, 18, 40, 44, 51, 56, 58). However, negative results in these animals (known to be exposed to *C. abortus* through vaccination and/or challenge) could also be due to a lack of sensitivity of the ELISA used, based on the detection of antibodies against major outer membrane proteins (MOMPs), considered to be less sensitive than tests based in polymorphic outer membrane proteins (POMPs) (9, 36, 44, 45).

As previously discussed, no significant impact of vaccination on the reproductive outcome was detected here. However, our results demonstrate that vaccination with the evaluated vaccine did have a beneficial effect on the weight gain of lambs born to vaccinated ewes (regardless the dose) during their first month of life. This effect, however, was not evident when overall weights recorded at 30 days of life or overall weight gain during those 30 days were compared. The protective effect is in agreement with a previous study reporting an effect of vaccination on lamb weight during their first weeks of life (4). However, the analytical approach used here allowed us to account for other likely confounders (such as type of pregnancy or weight at birth) and quantified the effect on lamb weight.

In conclusion, the present study did not find a significant effect of immunization with an inactivated vaccine on prevention of reproductive failure at standard or diluted doses. However, a beneficial effect of vaccination on lamb weight during the first month of life was found regardless the vaccination dose as evidenced by the association found between vaccination in the ewes and daily weight measured in the lambs, providing additional quantitative data that demonstrates the impact of *C. abortus* infection in a flock beyond the occurrence of reproductive failures. Further studies would be needed in order to determine the significance of our findings beyond the first month of age of the new-born lambs in terms of performance and health. In addition, our results highlight the usefulness of weight gain data for a full evaluation of vaccine efficacy.

## Author Contributions

TG-S: study design, coordination, supervision and performance of field and laboratory work, data analysis, and drafting manuscript. MP-S: field work, data analysis, and drafting manuscript. JS: study design, laboratory analysis overview, and critical review of manuscript. AN, PP, AD-G: field work, data collection, and laboratory analysis. NG: laboratory analysis and critical review of manuscript. JG and LD: study design and coordination, and critical review of manuscript. JA: study design and coordination, data analysis, drafting, and review of manuscript.

## Conflict of Interest Statement

The authors declare that the research was conducted in the absence of any commercial or financial relationships that could be construed as a potential conflict of interest.
