# Supplementary material for: Effect of Preventive Chlamydia abortus Vaccination in Offspring Development in Sheep Challenged Experimentally
Source: Front Vet Sci. 2016 Aug 25;3:67. doi: 10.3389/fvets.2016.00067 (PMC4997790; doi:10.3389/fvets.2016.00067)
Supplement: Supplementary file 1 [file Table_1.DOCX]

Supplementary Material

**Effect of preventive *Chlamydia abortus* vaccination in offspring development in sheep challenged experimentally**

**Teresa García-Seco^1^, Marta Pérez-Sancho^1, 2^, Jesús Salinas^3^, Alejandro Navarro^1^, Alberto Díez-Guerrier^4^, Nerea García^1^, Pilar Pozo^1^, Joaquín Goyache^1, 5^, Lucas Domínguez^1, 5^, Julio Álvarez^6*^**

*** Correspondence:** Corresponding Author: jalvarez@umn.edu

Supplementary table 1. ELISA qualitative results obtained in 51 ewes sampled after vaccination with an inactivated *Chlamydia abortus* commercial vaccine and challenged with a dose of 1 x 10^7^ inclusion-forming units of *C. abortus* strain AB7. 1: positive sample. 0: negative sample. NS: sample not available. a: day of administration the first vaccine dose subcutaneously. b: day of administration the second vaccine dose subcutaneously. c: day of experimental infection. d: beginning of reproductive events. †: died due to a distocic parturition.

|  | | **Days after vaccination** | | | | | | | | | | | | | | |
| --- | --- | --- | --- | --- | --- | --- | --- | --- | --- | --- | --- | --- | --- | --- | --- | --- |
| **Group** | **Ewe** | **0^a^** | **29^b^** | **41** | **56** | **69** | **83** | **96** | **103^c^** | **117** | **133** | **146^d^** | **161** | **168** | **174** | **182** |
| **C (Control)** | **1** | 0 | 0 | 0 | 0 | 0 | 0 | 0 | 0 | 1 | 1 | 1 | 1 | 1 | 1 | 1 |
|  | **2** | 0 | 0 | 0 | 0 | 0 | 0 | 0 | 0 | 1 | 1 | 1 | 1 | 1 | 1 | 1 |
|  | **3** | 0 | 0 | 0 | 0 | 0 | 0 | 0 | 0 | 1 | 1 | 1 | 1 | 1 | 1 | 0 |
|  | **4** | 0 | 0 | 0 | 0 | 0 | 0 | 0 | 0 | 0 | 0 | 0 | † | - | - | - |
|  | **5** | 0 | 0 | 0 | 0 | NS | 0 | 0 | 0 | 1 | 1 | 1 | 1 | 1 | 1 | 1 |
|  | **6** | 0 | 0 | 0 | 0 | NS | 0 | 0 | 0 | 1 | 1 | 1 | 1 | 1 | 1 | NS |
|  | **7** | 0 | 0 | 0 | 0 | NS | 0 | 0 | 0 | 1 | 1 | 1 | 1 | 1 | 1 | NS |
|  | **8** | 0 | 0 | 0 | 0 | NS | 0 | 0 | 0 | 1 | 1 | 1 | 1 | NS | 1 | NS |
|  | **9** | 0 | 0 | 0 | 0 | 0 | 0 | 0 | 0 | 1 | 1 | 1 | 1 | 1 | 1 | 1 |
|  | **10** | 0 | 0 | 0 | 0 | 0 | 0 | 0 | 0 | 1 | 1 | 1 | 1 | 1 | 1 | 1 |
|  | **11** | 0 | 0 | 0 | 0 | 0 | 0 | 0 | 0 | 1 | 0 | 1 | 0 | 1 | 0 | 0 |
|  | **12** | 0 | 0 | 0 | 0 | 0 | 0 | 0 | 0 | 1 | 0 | 1 | 1 | 1 | 1 | 0 |
|  | **13** | 0 | 0 | 0 | 0 | 0 | 0 | 0 | 0 | 1 | 1 | 1 | 0 | 1 | 1 | 0 |
|  | **14** | 0 | 0 | 0 | 0 | 0 | 0 | 0 | 0 | 1 | 1 | 1 | 1 | 1 | 1 | 0 |
|  | **15** | 0 | 0 | 0 | 0 | 0 | 0 | 0 | 0 | 0 | 0 | 0 | 0 | 0 | 0 | 0 |
|  | **16** | 0 | 0 | 0 | 0 | 0 | 0 | 0 | 0 | 0 | 0 | 0 | 0 | 0 | 0 | 0 |
|  | **17** | 0 | 0 | 0 | 0 | 0 | 0 | 0 | 0 | 1 | 0 | 0 | 0 | 0 | 1 | 0 |
|  | **18** | 0 | 0 | 0 | 0 | 0 | 0 | 0 | 0 | 1 | 1 | 1 | 1 | 1 | 1 | 1 |
| **DV (1/2 dose vaccine)** | **19** | 0 | 1 | 1 | 0 | 0 | 0 | 0 | 0 | 0 | 0 | 0 | 0 | 0 | 0 | 0 |
|  | **20** | 0 | 0 | 0 | 0 | 0 | 0 | 0 | 0 | 0 | 0 | 0 | 0 | 0 | 0 | 0 |
|  | **21** | 0 | 0 | 0 | 0 | 0 | 0 | 0 | 0 | 0 | 0 | 0 | 0 | NS | 0 | 0 |
|  | **22** | 0 | 0 | 0 | 1 | 1 | 1 | 1 | 1 | 1 | 1 | 1 | 1 | 1 | 1 | 1 |
|  | **23** | 0 | 0 | 0 | 0 | 0 | 0 | 0 | 0 | 0 | 0 | 0 | 0 | 0 | 0 | 0 |
|  | **24** | 0 | 0 | 0 | 0 | 0 | 0 | 0 | 0 | 1 | 1 | 1 | 1 | 1 | 1 | 1 |
|  | **25** | 0 | 1 | 1 | 0 | NS | 0 | 1 | 1 | 0 | 0 | 1 | 0 | NS | 0 | NS |
|  | **26** | 0 | 1 | 1 | 1 | 1 | 1 | 1 | 1 | 1 | 1 | 1 | 1 | 1 | 1 | 1 |
|  | **27** | 0 | 0 | 0 | 1 | 0 | 0 | 0 | 0 | 1 | 0 | 1 | 1 | 1 | 1 | 0 |
|  | **28** | 0 | 1 | 1 | 0 | 0 | 0 | 0 | 0 | 1 | 0 | 1 | 1 | 1 | 1 | 1 |
|  | **29** | 0 | 0 | 0 | 0 | 0 | 0 | 0 | 0 | 0 | 0 | 0 | 1 | 1 | 1 | 1 |
|  | **30** | 0 | 0 | 1 | 1 | 1 | 1 | 1 | 1 | 1 | 1 | 1 | 1 | 1 | 1 | 1 |
|  | **31** | 0 | 0 | 0 | 0 | 0 | 0 | 0 | 0 | 0 | 0 | 1 | 0 | 1 | 1 | 0 |
|  | **32** | 0 | 1 | 0 | 0 | 0 | 0 | 0 | 0 | 0 | 0 | 0 | 0 | 0 | 0 | 0 |
|  | **33** | 0 | 1 | 0 | 0 | 0 | 0 | 0 | 0 | 0 | 0 | 0 | 0 | 1 | 0 | 0 |
|  | **34** | 0 | 1 | 0 | 0 | 1 | 0 | 1 | 1 | 1 | 1 | 1 | 1 | 1 | 1 | 1 |
|  | **35** | 0 | 0 | 0 | 0 | 0 | 0 | 0 | 0 | 0 | 0 | 0 | 0 | 0 | 0 | 0 |
| **SV (Standard dose vaccine)** | **36** | 0 | 1 | 1 | 1 | 1 | 1 | 1 | 1 | 1 | 1 | 1 | 1 | 1 | 1 | 1 |
|  | **37** | 0 | 1 | 1 | 0 | 0 | 0 | 0 | 0 | 1 | 1 | 1 | 1 | 1 | 1 | 0 |
|  | **38** | 0 | 0 | 0 | 1 | 1 | 1 | 1 | 1 | 1 | 1 | 1 | 1 | 1 | 1 | 1 |
|  | **39** | 0 | 1 | 0 | 0 | 0 | 0 | 0 | 0 | 1 | 1 | 1 | 1 | 1 | 1 | 1 |
|  | **40** | 0 | 1 | 1 | 0 | NS | 0 | 1 | 1 | 0 | 0 | 0 | 0 | NS | 1 | NS |
|  | **41** | 0 | 0 | 0 | 0 | 0 | 0 | 0 | 0 | 0 | 0 | 0 | 0 | 0 | 0 | 0 |
|  | **42** | 0 | 1 | 1 | 1 | 1 | 1 | 1 | 1 | 1 | 0 | 1 | 1 | 1 | 0 | 1 |
|  | **43** | 0 | 0 | 0 | 0 | 0 | 0 | 0 | 0 | 0 | 0 | 0 | 0 | 0 | 0 | 0 |
|  | **44** | 0 | 0 | 0 | 0 | NS | 0 | 0 | 0 | 0 | 0 | 0 | 0 | 0 | 0 | 0 |
|  | **45** | 0 | 0 | 1 | 1 | 1 | 1 | 1 | 1 | 1 | 1 | 1 | 1 | 1 | 1 | 1 |
|  | **46** | 0 | 1 | 1 | 0 | 1 | 1 | 1 | 0 | 1 | 1 | 1 | 1 | NS | 1 | 1 |
|  | **47** | 0 | 0 | 0 | 1 | 1 | 1 | 1 | 0 | 1 | 1 | 1 | 1 | 1 | 1 | 1 |
|  | **48** | 0 | 1 | 1 | 0 | 0 | 0 | 0 | 0 | 1 | 1 | 1 | 1 | 1 | 1 | 0 |
|  | **49** | 0 | 0 | 1 | 0 | 0 | 0 | 0 | 0 | 0 | 0 | 0 | 0 | 0 | 1 | 0 |
|  | **50** | 0 | 1 | 1 | 1 | 1 | 1 | 0 | 1 | 1 | 1 | 1 | 1 | 1 | 1 | 1 |
|  | **51** | 0 | 0 | 0 | 0 | 0 | 0 | 0 | 0 | 0 | 0 | 0 | 0 | 0 | 0 | 0 |


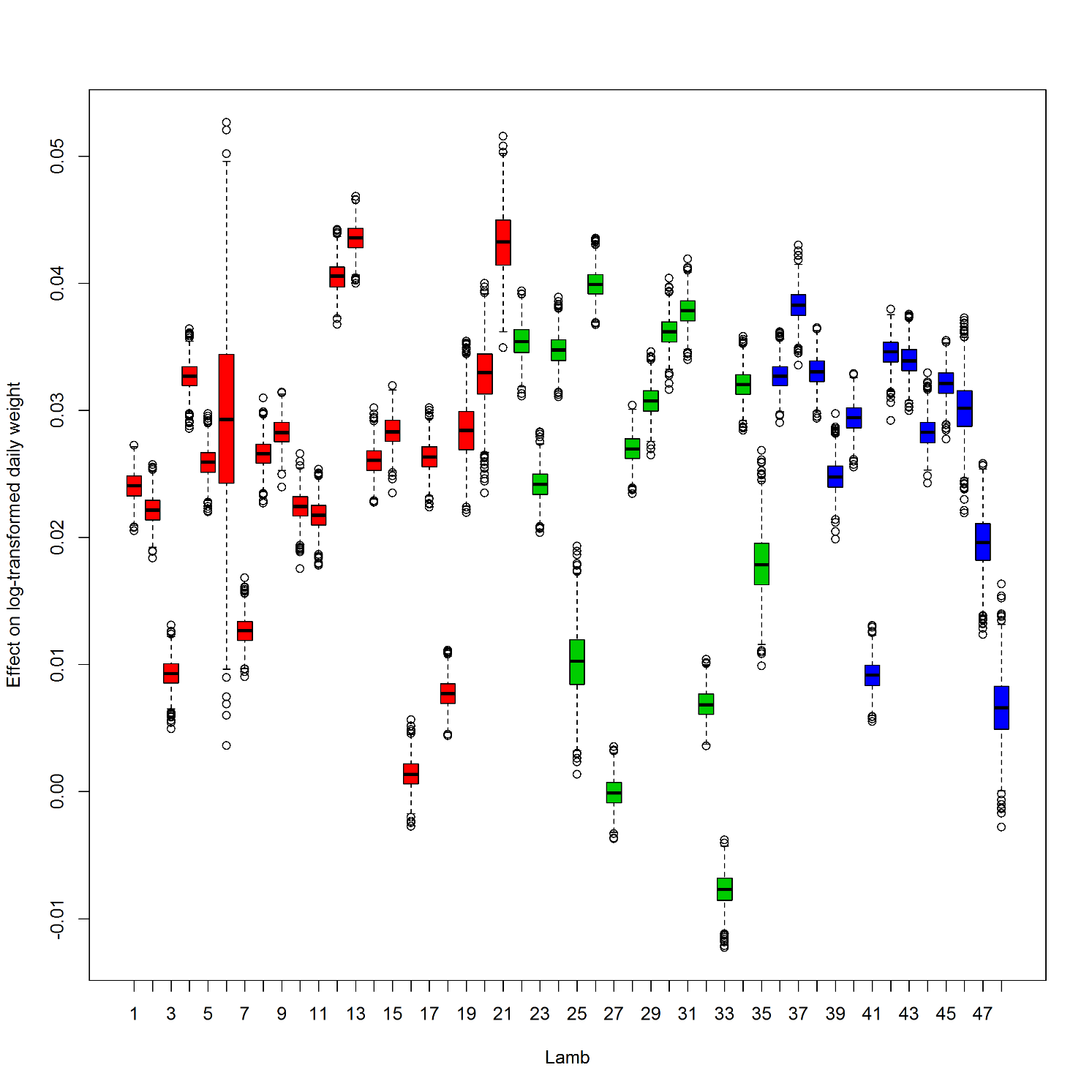


Supplementary figure 1. Boxplot of the animal-level random effect as determined in the model measuring the association between the log-transformed weight recorded in 48 lambs during their first 30 days of life and the lamb-related variables. Red: control Group C. Green: Group DV (1/2 dose). Blue: Group SV (standard dose).
